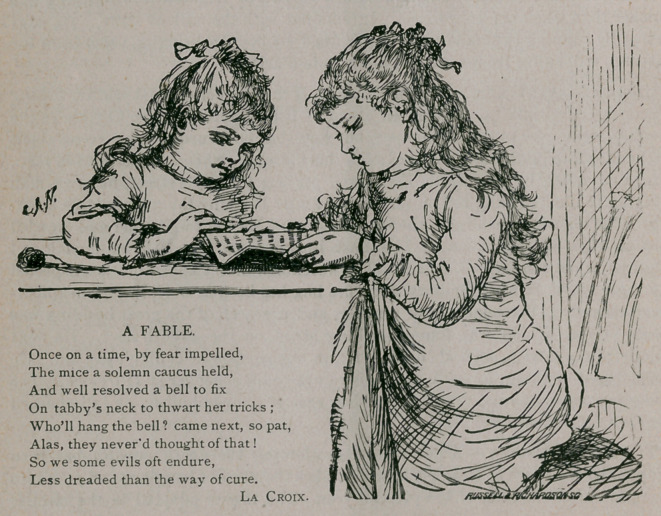# Miscellaneous

**Published:** 1888-08

**Authors:** 


### MISCELLANEOUS.

A Sorry Picture!—Church Masqueraders and Mountebanks who Scandalize the Church.—The Rev. S. Gifford Nelson, pastor of the Trinity Baptist Church, Brooklyn, preached from the above text on Sunday evening, May 13, in the course of which he used the following language:—"The number of those churchmembers who walk about in the daylight and in the night descend into the bottomless pit, would, if known, pass all belief. Thousands of men and women who are arrayed in gorgeous apparel are gangrenous with sensuality. The evil of licentiousness is growing at a frightful rate. We of the church have been lulled by the devil into a paltry sentimentality that fears to give offence to delicacy by denunciation of foul and lustful iniquities, and so the stalking horses of satan are roaming about in our churches. The church was scandalized by those who entered it for political, social and business advantages. The church itself was much to blame for this state of affairs. It bowed before men who would aid it with money and influence and turned its edifices into places for getting money by almost any means. Its ministers degraded religious sentiment by preaching sermons which would merely provoke laughter and amusement. Reform could come only through strict adherence to the principles taught us in the Bible.

It is generally accepted that brewers' grain is not a wholesome food for cows, and the milk furnished by them, when fed from it, is of an inferior quality, and not fit for habitual use, especially as food for infants.

It is true that the milk of cows who are worried or frightened will sour much quicker than when not so worried. Infants fed with the milk of cows worried or heated by running (which is sometimes caused by boys in bringing them from the pasture), will suffer from colic and often from diarrhœa.

Case of Somnambulism.—Joseph T. Walsh is the skipper on a Gloucester fishing schooner. He came up with a full cargo of fish yesterday. The Captain in the evening went over to the city proper, but says that he returned to his vessel about an hour before midnight. At about 12½ this morning, when an East Boston man, returning home by the South Ferry, was passing up Lewis street, he saw a man clinging to the lightning rod on the Boston Sugar Refining Company's large building. The man was then up to the seventh story. He went up higher and crawled into an eighth-story window. The East Boston man found the watchman and inquired what the exploit meant. The watchman didn't know, but he suspected the man might be an incendiary, for a fire bug has been at work in that neighborhood recently. Policemen were sent for, and a search of the great building was made. Capt. Walsh was found strolling about on the seventh floor. He went quietly to the station, and when asked for an explanation he couldn't explain. He remembered nothing, he said, after his return to his schooner at 11 o'clock. The police became convinced that it was a clear case of somnambulism, and discharged the Captain.—*New York Sun.*

The Death Penalty.—A new mode for enforcing the death penalty has been adopted by the State of New York, which takes the place of hanging, and goes into operation after the close of the present year. The means adopted is the electric shock, which is instantaneous and without conscious suffering or pain, and so far as these considerations have weight, the new system is an improvement upon the old. This or some change of the kind was recommended by Governor Hill in his first message to the General Assembly, and it has now become a law by his approval.

Lord Rosse's Big Telescope.—To an astronomer, of course, the chief interest of Birr Castle, Ireland, lies in the colossal telescopes, which were constructed by the father of the present Earl of Rosse nearly fifty years ago, and in his hands and those of his son have contributed so much to our knowledge of the nebulæ and to some branches of astronomical physics. There are three of them, all reflectors ; one 18 inches in diameter, which is mounted in a dome of its own, one 3 feet in diameter, and the "Leviathan," of 6 feet aperature and nearly 60 feet in length, incomparably the most immense of all astronomical instruments, though probably in real power such great refractors as the Pulkowa telescope and that of the Lick Observatory would over-match it. The smaller instruments have been pretty much reconstructed during recent years, and the 3 foot telescope especially as regards everything except the speculation, is far more the work of the present owner than of his father. Its equatorial mounting is of a pattern quite unique, and the arrangement by which the observer is enabled to reach the eye-piece is extremely ingenious. He stands in a sort of cage or basket which hangs from the arm of a crane that swings him around into the necessary position. The mounting of the great telescope has also received some really important improvements of late, but they are not very conspicuous, and in the main its general appearance is the same as when first erected 1842.—*Professor Young.*

A FABLE.

Once on a time, by fear impelled,  
The mice a solemn caucus held,  
And well resolved a bell to fix  
On tabby's neck to thwart her tricks ;  
Who'll hang the bell? came next, so pat,  
Alas, they never'd thought of that!  
So we some evils oft endure,  
Less dreaded than the way of cure.

La Croix.

## Figures and Tables

**Figure f1:**